# The prevalence of macrovascular complications among diabetic patients in the United Arab Emirates

**DOI:** 10.1186/1475-2840-6-24

**Published:** 2007-09-19

**Authors:** Fatma Al-Maskari, Mohammed El-Sadig, John N Norman

**Affiliations:** 1 Department of Community Medicine, Faculty of Medicine & Health Sciences, United Arab Emirates University. Al-Ain, PO Box: 17666, UAE

## Abstract

**Background:**

Diabetes Mellitus (DM) is a major public health problem in the UAE with a prevalence rate reaching 24% in national citizens and 17.4% in expatriates. The aim of this study was to determine the prevalence and risk factors of macrovascular complications among diabetic patients in the Al-Ain district of the United Arab Emirates (UAE).

**Methods:**

The study was part of a general cross-sectional survey carried out to assess the prevalence of diabetes (DM) complications among known diabetic patients in Al-Ain District, UAE. Patients were randomly selected during 2003/2004. Patients completed an interviewer-administered questionnaire carried out by treating doctors and underwent a complete medical assessment including measurement of height, weight, blood pressure and examination for evidence of macrovascular complications. A standard ECG was recorded and blood samples were taken to document fasting blood sugar, glycosylated haemoglobin (HbA_1C_) and lipid profile.

**Results:**

A sample of 513 diabetic patients was selected with a mean age of 53 years (SD ± 13.01). Overall, 29.5% of DM patients had evidence of macrovascular complications: 11.6% (95% CI: 8.8–14.4) of patients had peripheral vascular disease (PVD), 14.4% (95% CI: 11.3–17.5) had a history of coronary artery disease (CAD) and 3.5% (95% CI: 1.9–5.1%) had cerebrovascular disease (CVD). Of the total population surveyed 35% (95%CI: 30.8–39) had hypertension. The analysis showed that macrovascular complications in diabetic patients were more common among males, increased with age, were more common among hypertensive patients and its prevalence increased steadily with duration of DM.

**Conclusion:**

Our data revealed a significant association between hypertension and presence of macrovascular disease among diabetic patients. However, the risk of CAD in the UAE was relatively low compared to that seen in patients in other geographical settings. In addition, a lack of correlation between macrovascular disease and glycemic control among patients with DM was observed.

## Background

DM is known to be associated with a high risk of developing vascular complications which can lead to premature death and/or disability mainly by increasing the risk of myocardial infarction, stroke and peripheral vascular disease [1]. Patients with DM are two to four times more likely to develop cardiovascular disease than those in the general population and have a two to five time's greater risk of dying from these diseases [2].

Coronary and cerebrovascular diseases are reported to be two to three times more common in those with DM, and their associated mortality is also increased [1,3]. In the worldwide INTERHEART study of patients from 52 countries, diabetes accounted for 10% of the population attributable risk of first MI [4]. Transient ischaemic attacks are two to six times more common in DM patients and the risk of vascular dementia is also augmented [2]. The risk of peripheral vascular disease (PVD) in diabetics is four times higher [5,6] and is known to increase the risk of lower limb amputation by 15–40 times compared to the general population [2]. In Canada, 21% of diabetic patients were found to have heart disease and 25% of all cardiac surgery can be attributed to diabetes [6,7].

In addition to being a risk factor for cardiovascular disease in its own right, DM is associated with a higher prevalence of other risk factors such as hypertension and dyslipidaemia, which, in turn, have a more harmful effect in the presence of diabetes. Not only are these complications debilitating to the patient, but they are accompanied by a significant economic burden to the patient, family members, and the nation's health care budget.

The prevalence of macrovascular complications among DM patients in the UAE has not hitherto been documented. Clearly, such data are important, given that macrovascular complications account for approximately 50% of all deaths among DM patients in the industrialized countries [2,5]. Indeed, at present it is generally upheld that patients with DM should be treated as though they already have heart disease. Thus, it is pertinent to determine the magnitude of macrovascular complications among DM patients in the UAE. This is the first ever cross sectional analysis carried out to assess the prevalence of macrovascular complications in diabetic patients in the UAE. The complications documented were coronary artery disease (CAD), cerebrovascular disease (CVD) and peripheral vascular disease (PVD) using standard procedures of clinical history, physical examination, laboratory and clinical investigations.

## Methods

### Overall design

The study was part of a general cross-sectional survey of DM patients carried out to assess and establish the prevalence of DM complications including macrovascular complications among diabetic patients in the Al-Ain District, UAE. Al-Ain city is located in the interior of Abu Dhabi Emirate and constitutes its second largest city (approximately 400,000 population) out of total population of 4.2 millions in the UAE. 

### Subjects and setting

Multi-stage random sampling was used to select 8 primary health care centers (PHC) in Al-Ain district (out of 22 rural and urban health care centers) in addition to two diabetic clinics in the only two governmental hospitals in the district (Tawam and Al-Ain hospitals). Thus, our sampling frame included all UAE national and non-national DM patients (types 1 and 2) of all ages of both genders, attending any of the selected primary health care centers (PHC) for any reason and diabetic clinics at the two hospitals for follow up. Within these primary sampling units (PHC and hospital clinics), a systematic random sampling was carried out to select a sub-sample of patients to be approached for participation in the study. In the absence of a computerized database in the UAE every third diabetic patient was approached to participate during the study period. A sample size of 625 was calculated to give a standard error in the prevalence of macrovascular complications of less than 2%. In total, 600 patients were contacted by general practitioners and diabetologists, out of which, 513 patients (86%) agreed to enroll. The study was approved by the Ethical Committee of the Faculty of Medicine and Health Sciences of the UAE University. The data were collected between September 2003 and May 2004.

### Data collection

After receiving informed consent, known diabetic patients in Al-Ain were interviewed by the treating doctors at PHC and hospital clinics and information pertinent to their DM type, duration, compliance with treatment as assessed by doctors, associated complications and co-morbidity was collected. Additionally, blood pressure was measured by a nurse early in the morning and prior to drawing blood samples in the sitting position, using a standard mercury sphygmomanometer. Height was measured without shoes, and weight recorded while wearing indoor clothing. Body mass index (BMI) (weight in Kg, divided by height in meters squared) was calculated. The WHO (1977, 1979) classification for BMI was used to estimate the degree of obesity [8]. The WHO definition of hypertension [9] was used in this study: systolic blood pressure 160 mmHg or more and/or a diastolic blood pressure 95 mmHg or more, or on going treatment with antihypertensive drugs. A standard 12- lead electrocardiogram (ECG) was recorded for all patients. Fasting blood samples were taken to assess lipid profile, blood sugar and glycated hemoglobin (HbA_1C_) levels. Total lipid profile (total cholesterol (TC), high density lipoprotein (HDL), TC/HDL Ratio, low density lipoprotein (LDL) and triglycerides) were measured by a capillary tube whole blood method using the Cholesterol LDX lipid analyzer. Dyslipidaemia was taken to be present when the total cholesterol was > 5.60 mmol/L and/or triglycerides > 2.10 mmol/L, LDL > 3.4 mmol/L, and or HDL < 0.91 mmol/L [10]. Glycated hemoglobin (HbA_1C_) was measured using the Bayer DCA 2000+ analyzer and a value of less than 7% was taken to indicate good glycemic control. Urinary albumin concentration was measured by using semi-quantitative dry immuno chemical screening strips (Micral 11 ^® ^test strips (Roche diagnostic GmbH Mannheim Germany). Micral Tests were performed on first morning urine collections and a value of more than 20 μg/min was judged as pathological.

Macrovascular complications were ascertained using standard procedures. Coronary artery disease was identified by symptoms of definite angina pectoris or of definite past myocardial infarction or ECG changes consistent with previous myocardial infarction. Cerebrovascular disease was identified by a positive answer to the query "have you ever had a stroke?" and required confirmation by the interviewing physician. The physician ascertained the presence of peripheral vascular disease on physical examination when one or more foot pulses were judged absent, or if amputation and/or gangrene were present.

### Statistical analysis

The data were coded and processed on IBM compatible computers, using the Statistical Package for Social Sciences (SPSS) software (version 13). Descriptive analysis, using standard statistical methods was performed. Chi-square and Fisher exact tests were used to ascertain the association between macrovascular disease and clinical variables.

## Results

### Sociodemographic characteristics of the study population

A total sample (n = 513) of diabetic patients of both gender and from all nationalities (UAE nationals and expatriates) resident in the Al-Ain district of Abu Dhabi emirate was selected. Of those, 52% were males, 27% aged 60 years or above (mean age 53.3), 75% were UAE nationals and almost two thirds of patients were illiterate. Of the total sample 12.8% (95%CI: 11.0–14.6) were current smokers while 8.2% (95%CI: 6.7–9.7) were ex-smokers (Table 1).

**Table 1:** Baseline Characteristics of DM Patients in Al-Ain District, UAE, 2003–2004 (n = 513).

**Variable**	**Prevalence of DM**	
	**n**	**Percent (95% CI)**

**Sex**		
Male	264	51.5 (47.2–55.8)
Female	249	48.5 (44.2–52.8)
**Level of Education**		
Illiterate	318	62.8 (58.6–67.0)
Completed primary school	99	19.6 (16.1–23.1)
Completed secondary school	60	11.9 (9.1–14.7)
Completed university	29	5.7 (3.7–7.7)
**Age group (Years)**		
40 or less	81	15.8 (12.6–19.0)
41 – 49	137	26.8 (23–30.6)
50 – 59	154	30.1 (26.1–34.1)
60 or above	140	27.3 (23.4–31.2)
**Nationality group**		
UAE	382	74.6 (70.8–78.4)
Arabian Gulf Countries Citizens	54	10.5 (7.8–13.2)
Other Arabs countries	54	10.5 (7.8–13.2)
Asians	22	4.3 (2.5–6.1)
**Smoking**		
Current smoker	64	12.8 (11.0–14.6)
Ex smoker	41	8.2 (6.7–9.7)
**BMI Group**		
Under weight (< 18.5)	6	1.2 (0.2–2.2)
Normal weight (18.5–24.99)	113	22.5 (18.8–26.2)
Overweight (25–29.99)	195	38.8 (34.5–43.1)
Obese (> 30)	188	37.7(33.3–41.7)

### Clinical characteristics

Of the total sample, the majority (86%) had type 2 DM, 49% were diagnosed incidentally and most (79%) were known to have had the disease for ≥ 10 years. Of the total population, 35% (95%CI: 30.8–39) had hypertension and 76% were obese or overweight. The majority of patients (84%) partially managed their DM with oral hypoglycemic agents and 24% by insulin. Two thirds were not following any exercise regime as part of their management plan. HbA_1C _results showed that 37.6 (95%CI: 32.8–42.4) had good glycemic control and 62.4 (95%CI: 57.6–67.2) poor control. Dyslipidaemia, as indicated by an elevated total cholesterol, was present in 34.4% (95%CI: 30.0–38.8)) of patients and elevated triglycerides in 25.2% (95%CI: 21.1–29.3) (Table 2). The ratio of total cholesterol to HDL showed that 80% were at high risk of CAD. The ratio of LDL to HDL showed that 63% of the population was at risk of CAD.

**Table 2:** Clinical Characteristics of DM Patients in Al Ain District, UAE, 2003–2004 (n = 513).

**Variable**	**Prevalence of DM**	
	**n**	**Percent (95% CI)**

**Type of DM**		
Type 1	68	13.6 (10.6–16.6)
Type 2	431	86.4 (83.4–89.4)
**Mode of Diagnosis**		
Incidental	245	48.5 (44.1–52.9)
Screening	39	7.7 (5.4–10.0)
Symptomatic	221	43.8 (39.5–48.1)
**Family History of DM **		
Present	270	54.3 (49.9–58.7)
Absent	227	45.7(41.3–50.1)
**Duration of the DM**		
< 1 year	33	6.6 (4.4–8.8)
1–5 years	199	40.0 (35.7–44.3)
6–10 years	161	32.3 (28.2–36.4)
11–20 years	98	19.7 (16.2–23.4)
> 21 years	7	1.4 (0.4–2.4)
**Hypertension **		
Present	178	34.9 (30.0–38.8)
Absent	332	65.1(61.0–69.2)
**Dyslipidaemia **		
High total cholesterol level	152	34.4 (30.0–38.8)
Within reference range	290	65.5 (61.1–69.9)
**Triglycerides **		
High triglycerides level	105	23.9 (19.9–27.9)
Within reference range	334	76.1(72.1–80.1)
**Microalbuminuria**		
Present	276	61.2 (56.7–65.7)
Absent	175	38.8 (34.3–43.3)
**HAB**_**1C**_		
Good control	150	37.6 (32.8–42.4)
Poor control	249	62.4 (57.6–67.2)
**Coronary Artery Disease**		
Present	73	14.4 (11.3–17.5)
Absent	434	85.6(82.5–88.7)
**Cerebrovascular Disease**		
Present	18	3.5 (1.9–5.1)
Absent	490	96.5 (94.9–98.1)
**Peripheral Vascular Disease**		
Present	59	11.6 (8.8–14.4)
Absent	451	88.5 (85.6–91.2)

### Prevalence of macrovascular complications

Of the total sample surveyed, 11.6% (95%CI: 9.0–14.3) had PVD, 14.4% (95%CI: 11.3–17.5) had coronary artery disease and 3.5% had cerebrovascular disease (CVD) (Table 2).

The predictive risk factors for PVD, using univariate analysis were: male gender (p < 0.001), increasing age (p < 0.001), incidental DM diagnosis (p = 0.006) and duration of diabetes (p < 0.001). Presence of hypertension was a strong risk factor (p = 0.01).

The risk of CAD was more common among males (17%) than females (11%) (p = 0.04), steadily increased with increasing age (p = 0.04) and with disease duration (p = 0.002). CAD was also more common among type 1 DM patients (25%) compared to type 2 (12%) (p = 0.007). The risk of CAD was significantly higher among patients with hypertension (19%) compared to those without hypertension (12%) (p = 0.04).

The overall prevalence of CVD in the sample population was greater among males (5%) compared to females (2%) (p = 0.02), and increased with increasing age (p = 0.02). However, no significant association was found between CVD and duration of DM (p = 0.45). The risk of CVD was more common among patients with hypertension (7%) compared to those without hypertension (2%) (p = 0.001).

The prevalence of macrovascular disease showed no significant association with patients' nationality, level of education, BMI, dyslipidaemia, smoking or glycemic control.

## Discussion

Diabetes mellitus (DM) has long been recognized as a major public health problem with far reaching consequences, not only for its adverse health impact on individuals, but also for its economic burden on the health care system and society at large [11]. The International Diabetes Federation (IDF) in 2003 ranked the UAE's prevalence rates for type 2 DM and impaired glucose tolerance (IGT) as the second highest in the world (20% for DM and 26% for IGT) [2].

This study has shown an overall prevalence of macrovascular disease among diabetics of 29.5%: 11.6% had PVD, 14.4% had evidence of CAD and 3.5% were considered to have CVD. Hypertension was evident in 35% of the group; dyslipidemia in 34% and the ratio of total cholesterol to HDL indicated that 80% were at high risk of CAD, whilst the ratio of LDL to HDL confirmed that 63% were at high risk of developing CAD.

Compared to the rates reported by the WHO Multinational study [12] our rate for CAD was three times lower, our rate for CVD was consistent with the rates reported, but our rate for PVD was twice as high as the comparative rates. Compared to a study assessing complications among insulin-dependent DM patients in Sudan [13] our rates were relatively low for CAD (28%) but were consistent with the rates reported for PVD (10%) and CVD (4%). Also, our prevalence rate for CAD was consistent with the findings of a study in Finland [14], which reported a similar rate [16%] among type 2 DM patients after 6.3 years of follow up.

The differences in our rates of macrovascular complications among DM patients could be attributed to differences in study design, DM patients mix, and/or population characteristics. However, it is also possible that the relatively low rates of CAD observed in our study population are related to the fact that the majority of the sample populations, (82%) were recruited from primary health care centers and not from hospital set up.

The analysis of possible risk factors revealed a significant association between the presence of macrovascular disease and presence of hypertension among the surveyed diabetic population (p < 0.005). This is consistent with results from studies in other settings elsewhere. For example, in Spain, a large multicenter, outpatient clinics cross sectional population study, on hypertensive and type 2 DM patients, evaluating the risk of cardiovascular disease and renal damage using ECG-LVH, GFR and/or urinary albumin excretion, was able to establish an increased prevalence of cardiovascular disease in patients with hypertension and type 2 DM [15].

In Iran, a hospital based study of patients admitted with acute coronary events, established the prevalence of a significant increase of risk of coronary artery disease among diabetic patients compared to non diabetic patients (p < 0.005; OR = 2.4) and among hypertensive patients compared to normotensive patients (p < 0.005) [16].

In Germany, a large population based study on patients with type 2 DM, evaluating the relationship between atherosclerotic vascular disease (AVD) and metabolic syndrome (single and various combinations), using AVA/NHPLI definitions, showed an increased risk of AVD among diabetic patients (OR for men 1.38 and for women 1.67 respectively). Interestingly, the corresponding OR for hypertension as a single trait were 4.22 and 7.69 respectively; thus proving that hypertension is highly significantly associated with a higher prevalence of cardiovascular disease [17].

By contrast we were not able to detect any association between the presence of macrovascular disease and glycemic control as determined by the level of HbA_1C_. Our observation of a lack of correlation between macrovascular disease and glycemic control among patients with DM is not unique. Indeed many longitudinal studies were unable to establish such a link [[8,12] and [18]. The reason may relate to the multifactorial nature of macrovascular complications as reported in other studies [19].

Further, unlike the experience elsewhere [20], our study was unable to prove association between BMI, dyslipidaemia and cardiovascular disease in the study population; but that is not uncommon. For example, although Lubree et al. (2002) showed that increased levels of body fat, using the BMI, predicted an increased level of insulin resistance and other cardiovascular risk factors, even when compared to waist to hip ratio (WHR) [21], other sources suggest that the WHR is a more useful predictor of type 2 DM [22] and cardiovascular diseases [20,23]. More recently, it has been shown that the sagittal abdominal diameter (SAD) is the best predictor of cardiovascular risk and mortality in men [24], the efficacy of which has been further confirmed for women, following a recent study in Sweden evaluating the predictive role of SAD as a marker for CAD in the Swedish population [20]. In addition to the noted debate, a further restriction to our study was relating to the use of WHR, especially in women for cultural reasons, to further explore the risk of cardiovascular disease using other parameters.

The prevalence rate of CAD in our UAE diabetic patients was rather low compared to reported rates of studies in other geographical settings. However, this is not unique to the UAE population alone. Evidence in the literature suggests that variations in prevalence of heart diseases and diabetes occur even within the boundaries of the same country. For example in the US a recent study showed that heart disease deaths were highest in the eastern parts of the country and lowest in the west and Rocky Mountains region [25]. Another US based study also showed significant variations in the prevalence of DM between the north and south; with higher rates in the southern part of the country [26]. Among the factors considered and analyzed were the climatic variations and temperature; though, no clear evidence was possible to conclude [27]. Other studies considered ethnic differences to explain the variation in the risk of cardiovascular diseases in the population [27]. Among those was a US based study which concluded that substantial part of the risk associated with ethnicity can be attributed to socioeconomic status and geographical location rather than ethnicity [27].

The study had few limitations. Firstly, sampling from clinics might not be representative to patients or the general population as undiagnosed subjects might have been excluded. Secondly, the design was cross-sectional analysis, which makes it difficult to establish causal relationships with certainty. Thirdly, it is known that DM is notoriously under-diagnosed, and therefore while our sample probably adequately reflects macrovascular complications among diagnosed DM patients; it is likely that these findings are only reflecting the tip of the iceberg. Finally, the data from patients in Al-Ain may not typically represent patients in all UAE regions.

## Conclusion

DM in the UAE is commonly associated with macrovascular disease and hypertension. However, the risk of CAD in UAE was relatively low compared to that seen in patients in other geographical settings. In addition, a lack of correlation between macrovascular disease and glycemic control among patients with DM was observed. Greater awareness and attention to the macrovascular complications in patients with DM in our region is needed.

## Abbreviations

AVA/NHPLI-American Heart Association/National heart, Lung, and blood Institute.

AVD – Atherosclerotic Vascular Disease.

BMI – Body Mass Index.

CAD – Coronary Artery Disease.

CVD – Cerebro Vascular Disease.

DM – Diabetes Mellitus.

ECG – Electrocardiogram.

GFR – Glomerular Filtration Rate.

HDL – High Density Lipoprotein.

IDF – International Diabetes Federation.

IGT – Impaired Glucose Tolerance.

LDL – Low Density Lipoprotein.

LVH – Left Ventricular Hypertrophy.

MI – Myocardial Infarction.

OR – Odds Ratio.

PHC – Primary Health Care.

PVD – Peripheral Vascular Disease.

SAD – Sagittal Abdominal Diameter.

TC – Total Cholesterol.

TC/HDL – Total Cholesterol: High Density Lipoprotein.

UAE – United Arab Emirates.

WHO – World Health Organisation.

WHR – Waist to Hip Ratio.

## Competing interests

The authors declare that they have no competing interests.

## Authors' contributions

FAM conceived the need for the survey in the UAE, participated in its design, and contributed to the interpretation of the results. MES, participated in the design of the study and the data analysis. JNN contributed to formulation of research question, manuscript reviews and data interpretation. FAM and MES, JNN collaborated in writing up the manuscript. All authors read and approved the final manuscript.
